# Prevention of mother-to-child transmission in HIV audit in Xhosa clinic, Mahalapye, Botswana

**DOI:** 10.4102/phcfm.v6i1.609

**Published:** 2014-09-18

**Authors:** Stephane Tshitenge, Andre Citeya, Adewale Ganiyu

**Affiliations:** 1 School of Medicine, University of Botswana, Botswana; 2 Mahalapye District Health Management Team, Botswana

## Abstract

**Background:**

The Mahalapye district health management team (DHMT) conducts regular audits to evaluate the standard of services delivered to patients, one of which is the prevention of mother-to-child-transmission (PMTCT) programme. Xhosa clinic is one of the facilities in Mahalapye which provides a PMTCT programme.

**Aim:**

This audit aimed to identify gaps between the current PMTCT clinical practice in Xhosa clinic and the Botswana PMTCT national guidelines.

**Setting:**

This audit took place in Xhosa clinic in the urban village of Mahalapye, in the Central District of Botswana.

**Methods:**

This was a retrospective audit using PMTCT Xhosa clinic records of pregnant mothers and HIV-exposed babies seen from January 2013 to June 2013.

**Results:**

One hundred and thirty-three pregnant women registered for antenatal care. Twenty-five (19%) knew their HIV-positive status as they had been tested before their pregnancy or had tested HIV positive at their first antenatal clinic visit. More than two-thirds of the 115 pregnant women (69%) were seen at a gestational age of between 14 and 28 weeks. About two-thirds of the pregnant women (67%) took antiretroviral drugs. Of the 44 HIV-exposed infants, 39 (89%) were HIV DNA PCR negative at 6 weeks. Thirty-two (73%) children were given cotrimoxazole prophylaxis between 6 and 8 weeks.

**Conclusion:**

The PMTCT programme service delivery was still suboptimal and could potentially increase the mother-to-child transmission of HIV. Daily monitoring mechanism to track those eligible could help to close the gap.

## Abstrait

**Le contrôle de prévention de la transmission du VIH (PTME) de la mère à l'enfant à la clinique Xhosa de Mahalapye au Botswana**.

**Contexte:**

L’équipe de gestion sanitaire du district de Mahalapye (DHMT) procède à des contrôles réguliers pour évaluer le niveau des services donnés aux patients, tels que le programme de prévention de la transmission du VIH de la mère à l'enfant (PTME). La clinique Xhosa est l'un des établissements à Mahalapye qui offre un programme PTME.

**Objectif:**

Cette évaluation avait pour but d'identifier l’écart entre les pratiques cliniques PTME actuelles à la clinique Xhosa et les directives nationales du PTME du Botswana.

**Lieu:**

Cette évaluation a eu lieu à la clinique Xhosa dans le village de Mahalapye, dans le District central du Botswana.

**Méthodes:**

Il s'agissait d'une rétrospective basées sur les rapports du PTME de la clinique Xhosa des mères enceintes et des bébés exposés au virus VIH examinés de janvier à juin 2013.

**Résultats:**

Sur les cent tente-trois femmes enceintes inscrites pour les soins prénataux, vingt-cinq (19%) savaient leur état séropositif car elles avaient été testées avant leur grossesse ou avaient été testées séropositives lors de leur première visite prénatale à la clinique. Plus des deux-tiers des 115 femmes enceintes (69%) avaient été examinées pendant la gestation entre 14 et 28 semaines. Environ les deux-tiers des femmes enceintes (67%) prenaient des médicaments antirétroviraux. Parmi les 44 enfants exposés au VIH, 39 (89%) étaient séronégatifs par test d'ADN-PCR à six semaines. Trente-deux enfants (73%) avaient reçu un traitement prophylactique au cotrimoxazole entre 6 et 8 semaines.

**Conclusion:**

Le service du PTME était encore sous-optimal et pourrait augmenter la transmission du VIH de la mère à l'enfant. Un mécanisme de contrôle quotidien et de dépistage des personnes concernées pourrait permettre de réduire l’écart.

## Introduction

### Background

Botswana's high HIV burden is an immediate threat to child survival as children born with HIV are vulnerable to both disease and death.^1^ The introduction of the Prevention of Mother-to-Child Transmission (PMTCT) programme has resulted in a significant reduction in the number of children born with HIV.

It has been a decade since the PMTCT programme was launched in the country. In this time, the spread of HIV from mother-to-child has decreased from about 40% to 4%.^2^ The Botswana government aims to reduce mother-to-child transmission to less than 1% by 2016.^3^ The Botswana PMTCT guidelines state that: (1) all HIV-positive pregnant women with a CD4+ cell count above 350 cells/mm3 should be initiated on triple antiretroviral prophylaxis (TAP) at 14 weeks; (2) all HIV-positive pregnant women with a CD4+ cell count below 350 cells/mm^3^ or who are at the World Health Organization (WHO) clinical stage 3–4 should be initiated on highly-active antiretroviral therapy (HAART), regardless of their gestational age; (3) all HIV-exposed infants should be initiated on cotrimoxazole (CTX) prophylaxis at six to eight weeks.^1^

The PMTCT programme is run in all health facilities across the country and has been integrated into antenatal care (ANC) as well as maternity and post-natal care. The Mahalapye district health management team (DHMT) conducts regular audits to evaluate the standard of services (such as PMTCT) that are delivered to patients. Xhosa clinic is one of the facilities in Mahalapye DHMT that provide the PMTCT programme.

### Aim and objectives

This audit aimed to identify gaps between the current PMTCT clinical practice in Xhosa clinic and the Botswana PMTCT national guidelines. The objectives were:

1. to determine the HIV status of pregnant women at their first ANC visit
2. to determine the proportion of pregnant women who booked early (before 28 weeks) for ANC appointments
3. to establish whether HIV-positive pregnant women with a CD4+ cell count above 350 cells/mm^3^ were initiated on TAP at 14 weeks
4. to establish whether HIV-positive pregnant women with a CD4+ cell count below 350 cells/mm^3^ or in WHO clinical stage 3–4 were initiated on HAART, regardless of their gestational age
5. to determine the HIV results of HIV-exposed infants at 6–8 weeks
6. to establish whether all HIV-exposed infants were initiated on CTX prophylaxis at 6–8 weeks.

## Research method and design

### Setting

Xhosa clinic is one of the 45 health facilities in Mahalapye, an urban village in the Central District of Botswana. The clinic provides only outpatient services and is one of the main antiretroviral (ARV) therapy service providers in the Mahalapye health district.

### Audit design

This was a retrospective audit using PMTCT Xhosa clinic registers, namely the ANC register and the Baby PCR (polymerase chain reaction) testing register.

### Sample

All pregnant women enrolled on PMTCT services and all HIV-exposed infants aged 6–8 weeks seen at Xhosa clinic between January 2013 to June 2013 were included in the study.

### Data collection and analysis

All ANC records of pregnant mothers and all records of Baby PCR tests of HIV-exposed babies from 01 January to 30 June 2013 were included. The following data were collected: gestational age, pregnant mother's HIV test at the first visit, CD4+ results, ARV treatment and HIV-exposed infants on CTX. All data collected from records of HIV-exposed babies were included. The standard of PMTCT patient management was adopted from the Botswana PMTCT and the Botswana Safe Motherhood Initiative guidelines.^1, 3^ Data were fed into a spreadsheet for analysis. Descriptive statistics such as frequencies were used to analyse the pooled data and the results were presented in graphics (bar charts) and tables. The audit team reported their findings to the Mahalapye DHMT.

### Ethical considerations

The clinical audit was approved by the Mahalapye DHMT (reference PBRS/2014). No patient identifier information (e.g. name, clinic number, address) was used. Access to the records was restricted to the audit team and information collected was stored securely in an electronic file with password. The electronic file was destroyed after completion of the audit. Information gathered during the audit process were only used for purposes of the audit. Only the summarised data to allow future comparison with any re-audit were kept. The audit in no way intended to victimise any particular person.

## Results

**FIGURE 1:**
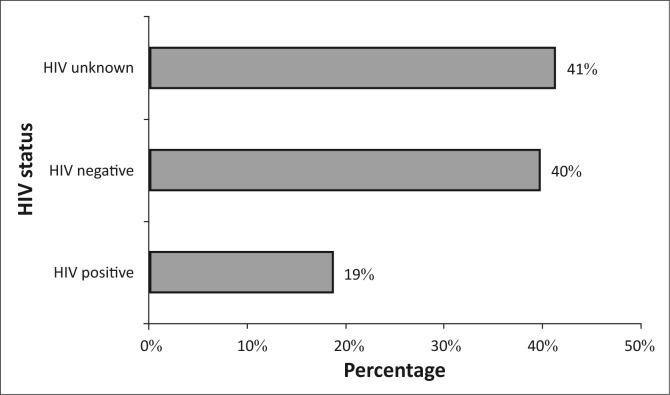
Bar chart demonstrating proportion of HIV status in women at their first antenatal care visit, Xhosa clinic, January 2013 to June 2013.

One hundred and thirty-three pregnant women registered for ANC from January 2013 to June 2013. Twenty-five (19%) knew their HIV-positive status as they had been tested before their pregnancy or had tested HIV-positive at their first ANC visit (Figure 1). The majority of the women either did not know their HIV status (*n* = 55; 41%) or tested HIV negative (*n* = 53; 40%) at their first ANC visit.

**FIGURE 2:**
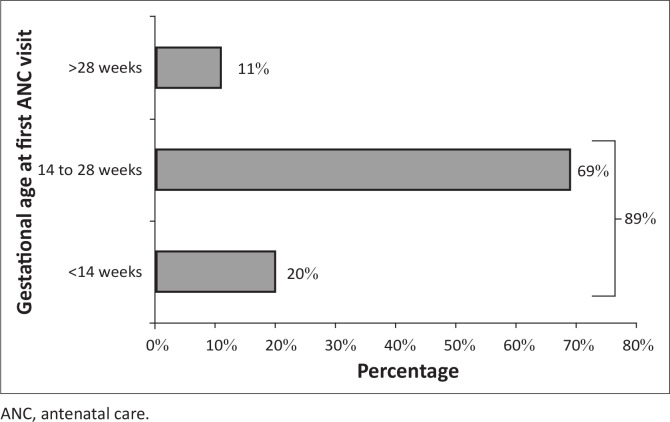
Bar chart demonstrating gestational age of pregnant women at first antenatal care visit, Xhosa clinic, January 2013 to June 2013.

Eighteen of the 133 pregnant women did not have their gestational age recorded in the register, but the gestational age of the majority of the remaining 115 women (*n* = 102; 89%) was before 28 weeks at their first antenatal visit (Figure 2). More than two-thirds of the 115 pregnant women (*n* = 79; 69%) were seen at a gestational age of between 14 and 28 weeks; whilst only 23 (20%) were seen at a gestational age of below 14 weeks. Only 13 of the pregnant women (11%) booked their first ANC visit after 28 weeks of gestation.

**FIGURE 3:**
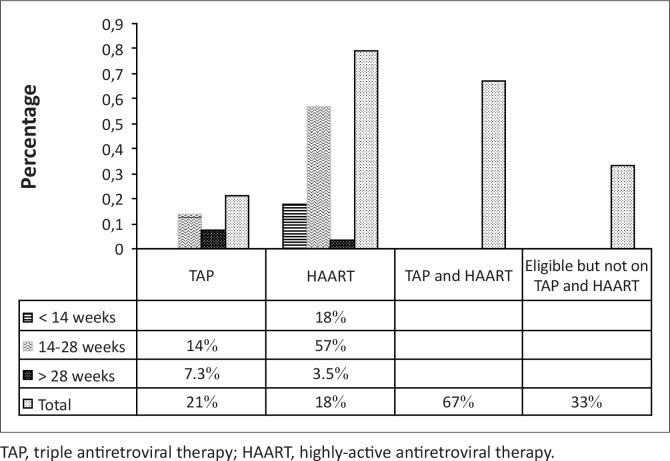
Bar chart demonstrating eligible patients on triple antiretroviral therapy or highly-active antiretroviral therapy versus eligible patients not initiated on treatment.

About two-thirds of the pregnant women (*n* = 45; 67%) took ARV drugs as TAP or HAART; whilst 33% (*n* = 22) of the pregnant women were eligible, but were not initiated on ARV drugs (Figure 3). The majority of those on ARV drugs took HAART (*n* = 52; 78%).

**FIGURE 4:**
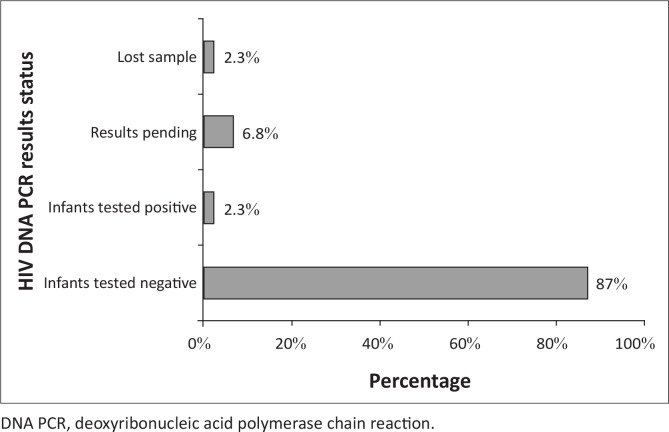
Bar chart demonstrating results of HIV DNA PCR at 6–8 weeks of HIV-exposed infants, Xhosa clinic, January 2013 to June 2013.

**FIGURE 5:**
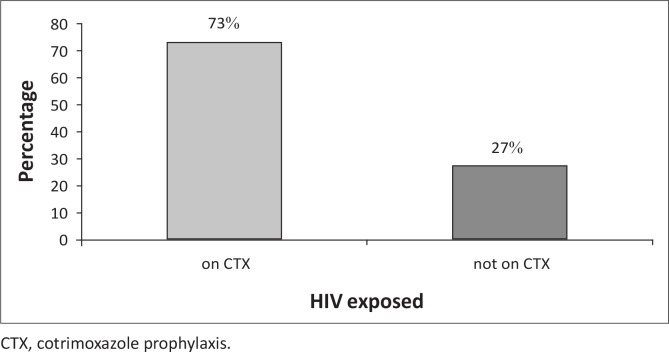
Bar chart demonstrating HIV-exposed infants given cotrimoxazole prophylaxis from 6–8 weeks, Xhosa clinic, January 2013 to June 2013.

The Xhosa clinic Baby PCR testing register indicated that 44 HIV-exposed infants were reviewed at ages 6 and 8 weeks during the study period. Out of the 44 HIV-exposed infants, 39 (87%) were HIV DNA (proviral HIV) PCR negative at six weeks, one (2.3%) was HIV DNA PCR positive, three (6.8%) results were still pending and one (2.3%) sample had been lost (Figure 4). Thirty-two (73%) children were given CTX prophylaxis between 6 and 8 weeks; whilst 12 (27%) of the HIV-exposed infants were not initiated on CTX prophylaxis (Figure 5).

## Discussion

This audit was part of the routine monitoring of the service delivery to our patients. In our audit, the proportion of the HIV-positive pregnant women (19%) was far from the HIV prevalence of 32% amongst ANC clinic attendees in Botswana.^1, 4, 5^ Close to half of pregnant women (41%) did not know their HIV status at the first visit. One may argue that the lower proportion of HIV-positive pregnant women from our audit, compared with the national one, did not reflect the true picture as a significant number of pregnant women with unknown HIV status contributed to the total.

The majority of pregnant women (87%) booked for their first ANC visit either before 14 weeks or between 14 and 28 weeks, which implied that the majority of these clients came on time to seek appropriate care.

The majority of pregnant women (67%) on ARV drugs were on a HAART regimen. The criteria for initiation of HAART are when the patient's CD4+ count is below 350 cells/mm^3^ and/or when the patient is classified as being at WHO clinical stage 3 or 4.^1, 6, 7^ The high proportion of pregnant women on HAART could suggest that there was good collaboration between the infectious disease centre clinic (IDCC) and the ANC clinic.

The proportion of HIV-positive pregnant women who were eligible for TAP or HAART, but not yet initiated on this regimen, was significant (33%). The PMTCT guidelines recommend that all eligible pregnant women should be given appropriate service. Poor documentation and inadequate monitoring could contribute to this inadequate service delivery. The transmission of HIV from mother to newborn increases in the absence of in utero PMTCT intervention.^8, 9^ It was estimated that during the period where PMTCT intervention in utero was absent in Botswana, the transmission estimates each year ranged from 684 to 1367.^1^

The majority of HIV-exposed infants (89%) tested negative. Only one (2.3%) HIV DNA PCR result was positive. This proportion was below the national proportion of 4% and the global target of 5% by 2015,^1, 2, 10^ but only a small number of children were tested. About three-quarters of the HIV-exposed infants (73%) were given prophylaxis between six and eight weeks. More effort should be put into covering the 27% of infants who did not receive appropriate services.

### Recommendations

The Mahalapye DHMT healthcare providers in Xhosa clinic should encourage the general public and patients to test for HIV in order to reduce the proportion of people who do not know their HIV status. Mahalapye DHMT should conduct health facility and community talks in order to educate mothers regarding early ANC booking. Daily monitoring to track those eligible for TAP, HAART and CTX should be implemented in the Xhosa clinic in order to reduce the proportion of eligible women and HIV-exposed children who are not on appropriate drugs. The effort to improve the current clinical practice with regard to PMTCT intervention during pregnancy and care of exposed babies could include more home care visits. More emphasis should be put to record keeping. We also recommended regular audits of clinical practice in order to measure the services provided to clients.

## Conclusion

The findings of this audit showed that there are gaps between the clinical practice in Xhosa clinic with regard to its PMTCT programme and the standard set by Botswana PMTCT guidelines. The PMTCT programme service delivery was still suboptimal with regard to the administration of ARV to HIV-positive pregnant women and in the provision of CTX prophylaxis to HIV-exposed infants. The audit was conducted in one institution in Mahalapye DHMT, so the findings cannot be generalised to the whole region.
